# ROCK inhibition with Fasudil induces beta-catenin nuclear translocation and inhibits cell migration of MDA-MB 231 human breast cancer cells

**DOI:** 10.1038/s41598-017-14216-z

**Published:** 2017-10-20

**Authors:** Fabiana Sélos Guerra, Ramon Guerra de Oliveira, Carlos Alberto Manssour Fraga, Claudia dos Santos Mermelstein, Patricia Dias Fernandes

**Affiliations:** 1 Universidade Federal do Rio de Janeiro, Instituto de Ciências Biomédicas, Laboratório de Farmacologia da Dor e da Inflamação, Rio de Janeiro, Brazil; 2 Universidade Federal do Rio de Janeiro, Instituto de Ciências Biomédicas, Laboratório de Avaliação e Síntese de Substâncias Bioativas (LASSBio®), Rio de Janeiro, Brazil; 3 Universidade Federal do Rio de Janeiro, Instituto de Ciências Biomédicas, Laboratório de Diferenciação Muscular, Rio de Janeiro, Brazil

## Abstract

Tumor aggressiveness is usually associated with metastasis. MDA-MB 231, a triple-negative breast cancer (TNBC), is an aggressive type of breast cancer and associated with early metastasis. The Rho/ROCK pathway is a key regulator of cell motility involving cytoskeleton regulation through stabilization of actin filaments and stress fiber formation. In this study we show that Fasudil, a ROCK inhibitor, inhibited the migration of MDA-MB 231 and A549 cells, without altering the viability of these cells at the concentration of 10 μM, modified tumor cell morphology, with disorganization of stress fibers and promotes activation of the canonical-Wnt/beta-catenin pathway. Therefore, Fasudil present a promising approach to the prevention of breast cancer metastasis through a different mechanism of action from the well-known one.

## Introduction

Breast cancer occupies the highest incidence rate among all cancers in females^1^. The heterogeneous nature of breast cancer has implications for biological behaviour, responses to treatment and prognosis. The ability of cancer cells to undergo invasion and migration is a prerequisite for tumour metastasis.

MDA-MB 231, a triple-negative breast cancer (TNBC), is an aggressive type of breast cancer and associated with early metastasis, drug resistance, and poor patient survival, which do not express estrogen receptor α (ERα), progesterone receptor (PR) and human epidermal growth factor receptor 2 (HER2). Patients with TNBC cannot benefit from the currently available endocrine and anti-HER2 therapies and have a high risk of recurrence and exhibits poor prognosis^2^. In this regard, it is necessary to further investigate the molecular pathogenesis of TNBC and to explore novel treatments of TNBC patients.

Rho are small GTPases that play important roles in many dynamic cellular processes, such as regulation of focal adhesion, actomyosin contraction, and cell motility^3^. Rho GTPases are expressed in three main isoforms, Rho-A, B and C, and the most important effector systems that are part of the signalling cascade of Rho-A are mDia and Rho-associated protein kinase (ROCK)^4^. ROCK is a serine threonine kinase modulating several critical cellular processes, such as actin cytoskeleton organization, apoptosis, reactive oxygen species formation, cell migration and adhesion. In mammalians, two highly homologous isoforms, ROCK1 and ROCK2 has been identified. While ROCK1 is primarily expressed in non-neuronal tissues, ROCK2 is preferentially detected in the brain, spinal cord and muscle^5^. These two isoforms share common structural features, such as an amino terminal kinase domain, a mild coiled-coil containing the Rho binding domain (RBD), and a cysteine rich domain (CRD) within the pleckstrin homology (PH) motif^6^. Both ROCK1 and ROCK2 share an overall 65% homology in their amino-acid sequence and 92% in their kinase domains. ROCK has several phosphorylation substrates, including myosin light chain (MLC), myosin light chain phosphatase (MLCP), LIM kinase (LIMK), all of which are involved in cytoskeleton regulation through stabilization of actin filaments and stress fiber formation^7^.

The Wnt signaling pathway is an evolutionarily conserved pathway that regulates crucial aspects of cell fate determination, cell migration, cell polarity, neural patterning and organogenesis during embryonic development. Perturbation of Wnt signaling with aberrant expression of Wnt factors, their receptors, or downstream signaling molecules may lead to the development of several human cancers^8^. Recently our group demonstrated that the disorganization of cholesterol enriched-lipid rafts leads to Wnt signaling resulting in reduced tumor cells migration^9^.

For the design of rational therapies, it is crucial to understand mechanisms that underlie the metastatic behaviour of TNBC cells and to characterise high risk metastasis. Recent studies identify ROCK as a promising candidate for a therapeutic target that could treat patients with highly metastatic cancer^10^. However, the function of ROCK particularly during the migration of TNBC cells is unclear, which hampers the precise interpretation of this target. Here, we show that Fasudil, a ROCK-inhibitor, induces a non-migratory phenotype in MDA MB 231 cells, with disorganization of stress fibers and activation of the canonical-Wnt/beta-catenin pathway. The collection of our data identifies a TNBC-specific mechanism of ROCK and beta-catenin and demonstrates the relevance of a cell-type specific background for the cancer-type-specific role of a protein kinase.

## Results

### Cell viability

**Figure 1:**
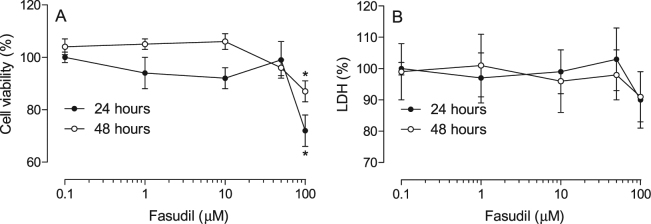
Effects of Fasudil in cell viability after 24 and 48 h of incubation. MDA-MB 231 cells were incubated with different concentrations of Fasudil for 24 or 48 h. Cell viability was analysed using a MTT (in **A**) or LDH (in **B**)-based methods (described in methods section). Results are represented as media ± standard deviation (n = **4**) of independent experiments. Statistical analyses were performed by analysis of variance followed by Newman-Keuls post-test. *p < 0.05 when compared with control group.

To evaluate the effects of Fasudil on cell viability we performed a MTT-based and a lactate desidrogenase (LDH)-based assay. We analysed the viability of the cells after 24 and 48 h of treatment with increasing concentrations of Fasudil (0.1, 1, 10, 50 and 100 µM). The results of the MTT assay showed that from 0.1 to 50 µM of Fasudil cell viability was not altered after 24 or 48 h of treatment, whereas 100 µM of Fasudil reduced cell viability in both 24 h (25% reduction) and 48 h (10% reduction) of incubation in the MTT assay (Fig. 1A). When analysing LDH liberation by cells incubated with same concentrations of Fasudil we observed that even higher concentration (100 µM) of Fasudil did not induce liberation of the enzyme (Fig. 1B). To rule out a possible cell-specific effect we performed the same assays using a lung tumor cell line (A549). In this context, no alteration was observed in the release of LDH nor MTT conversion (data not shown).

### Cell migration quantified by the cell-based scratch assay

**Figure 2:**
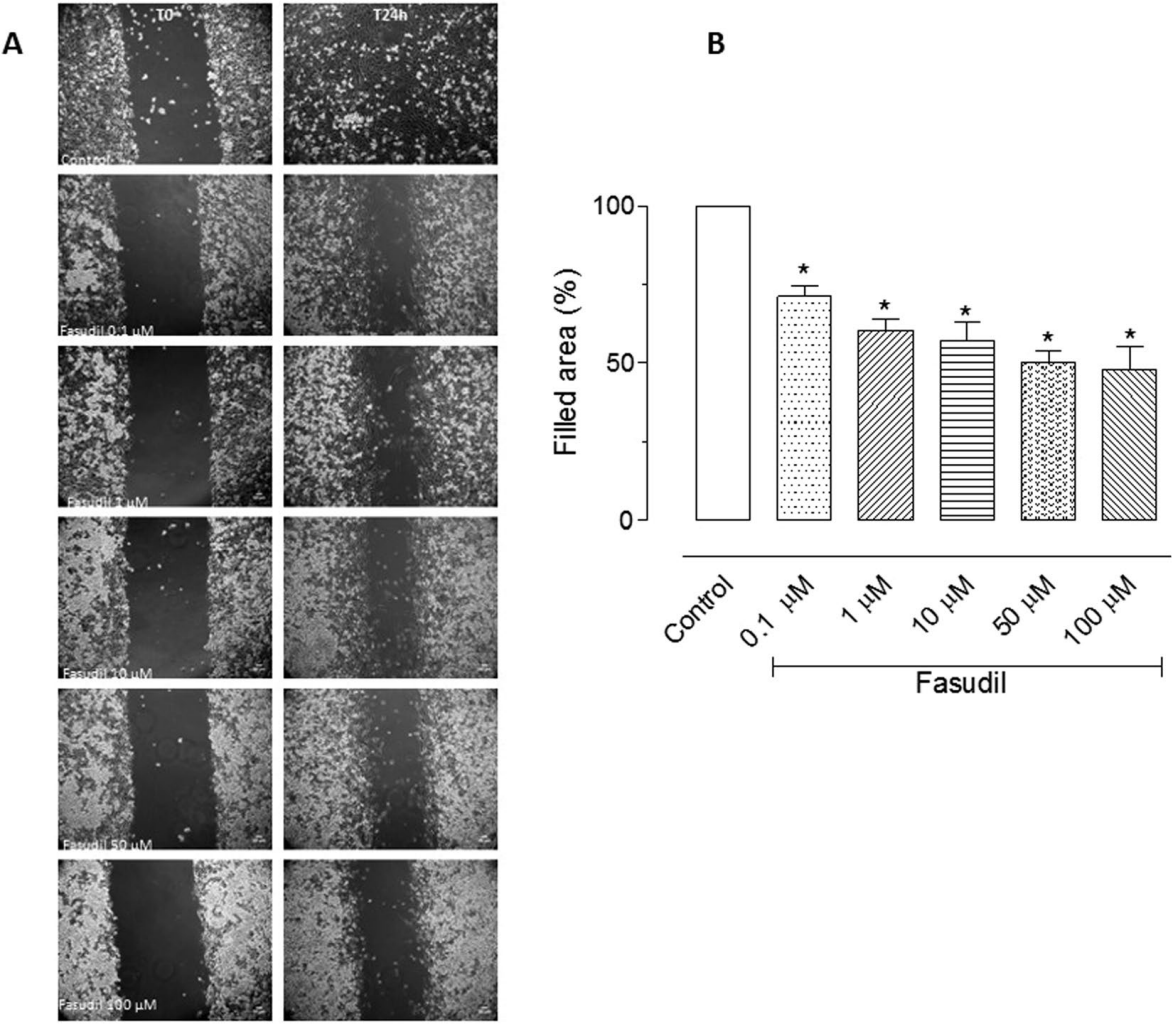
Effect of Fasudil in migration of MDA-MB 231 cells. Cells were cultured up to 90–100% confluence and then scratched wound lines were created. After 24 h of incubation with different concentrations of Fasudil, images were obtained by using phase-contrast microscopy. Filled area composed by cells that migrated was calculated using ImageJ software. Results are represented as media ± standard deviation (n = 4) of independent experiments. Statistical analyses were performed by analysis of variance followed by Newman-Keuls post-test. *p < 0.05 when compared with control group. Scale bars represent 50 μm.

To analyse whether Fasudil could alter cell migration, we performed a cell-based scratch assay followed by the quantification of the wounded area. Cells were cultured up to 90–100% confluence and then scratched wound lines were created with a micropipette tip. Some cultures were treated for 24 h with Fasudil (0.1, 1, 10, 50 or 100 µM). Interestingly, untreated cells nearly covered the scratched areas of the dish in 24 h, whereas in Fasudil-treated cultures relatively large empty areas were visible in the culture dishes after 24 h of treatment in all tested concentrations (Fig. 2A and B). Empty areas varied from 25% to 50%, as compared to control untreated cultures (Fig. 2A and B).

**Figure 3:**
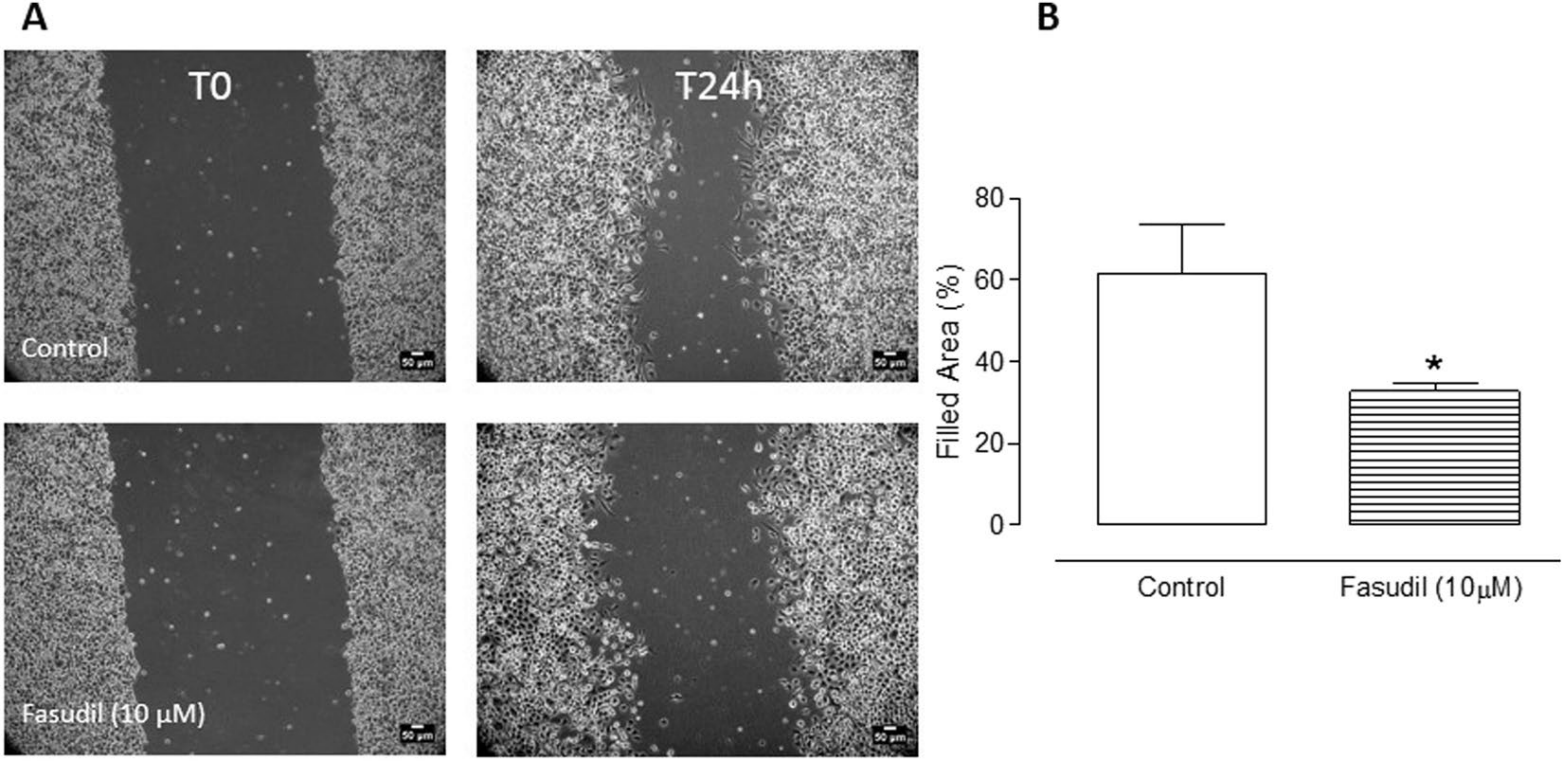
Effect of Fasudil in migration of A549 cells. Cells were cultured up to 90–100% confluence and then scratched wound lines were created. After 24 h of incubation with 10 µM Fasudil, images were obtained by using phase-contrast microscopy. Filled area composed by cells that migrated was calculated using ImageJ software. Results are represented as media ± standard deviation (n = 4) of independent experiments. Statistical analyses were performed by analysis of variance followed by Newman-Keuls post-test. *p < 0.05 when compared with control group. Scale bars represent 50 μm.

To discard a possible cell-specific effect of fasudil against MDA-MB231 cell we decided to further analyse the effects of the drug against a lung tumor cell line (A549). In this regard, cells were incubated with 10 µM of Fasudil. Although we did not observe a complete coverage of the scratched areas of the dish in control cells after 24 h incubation, Fasudil-treated groups caused a reduction in coverage area with almost 50% empty areas (Fig. 3A and B).

### Fasudil-treatment induces changes in cell morphology

**Figure 4:**
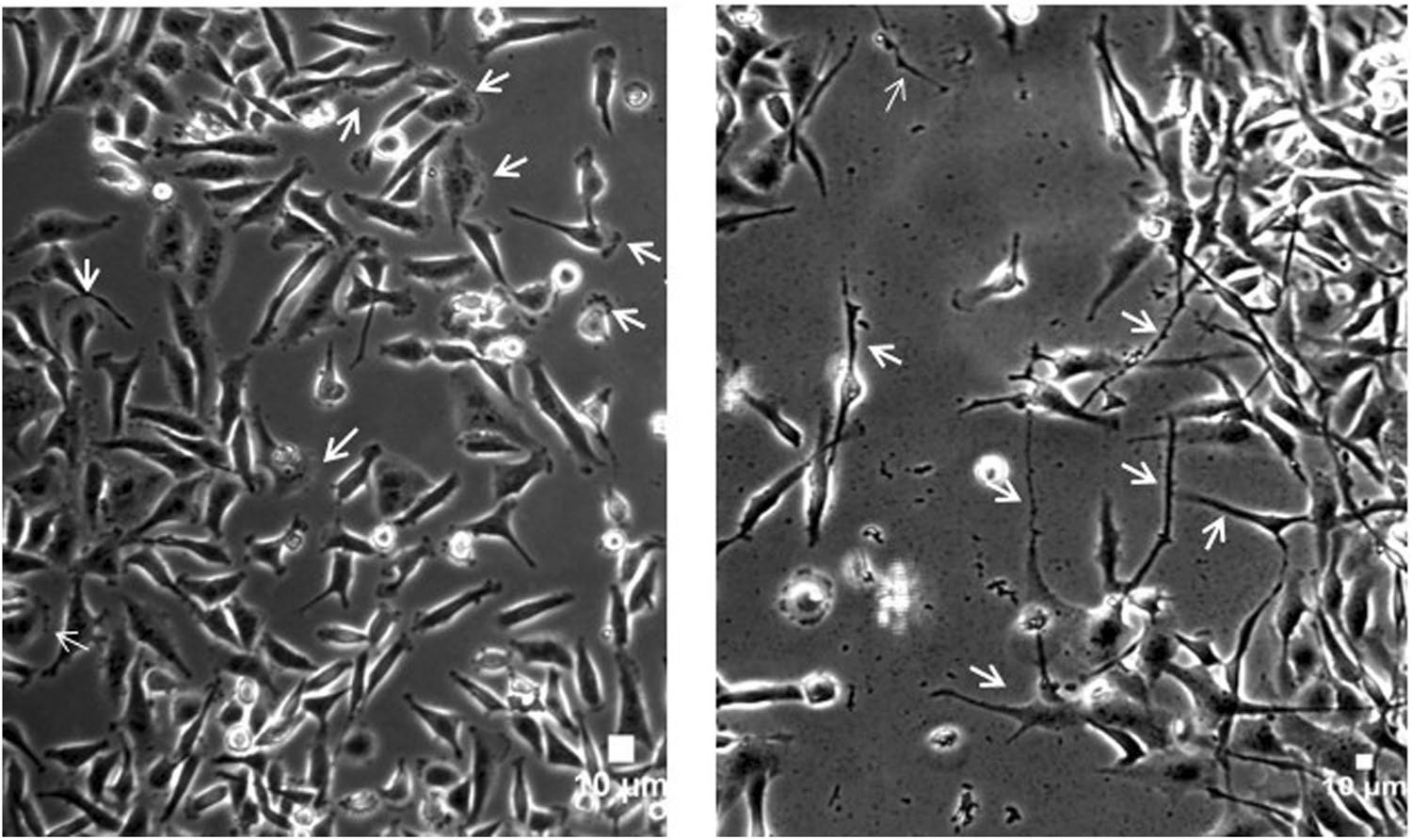
Fasudil-treatment induces changes in cell morphology. MDA-MB 231 cells were grown for 24 h cultures and treated with Fasudil (10 µM) for 24 h. Cells were analysed by phase contrast microscopy. Note that control cells (**A**) display many ruffled membranes and lamellipodia (arrows) whereas in Fasudil-treated cells (**B**) membrane protrusions were less prominent and most of the cells are spindle-shaped (arrows).

**Figure 5:**
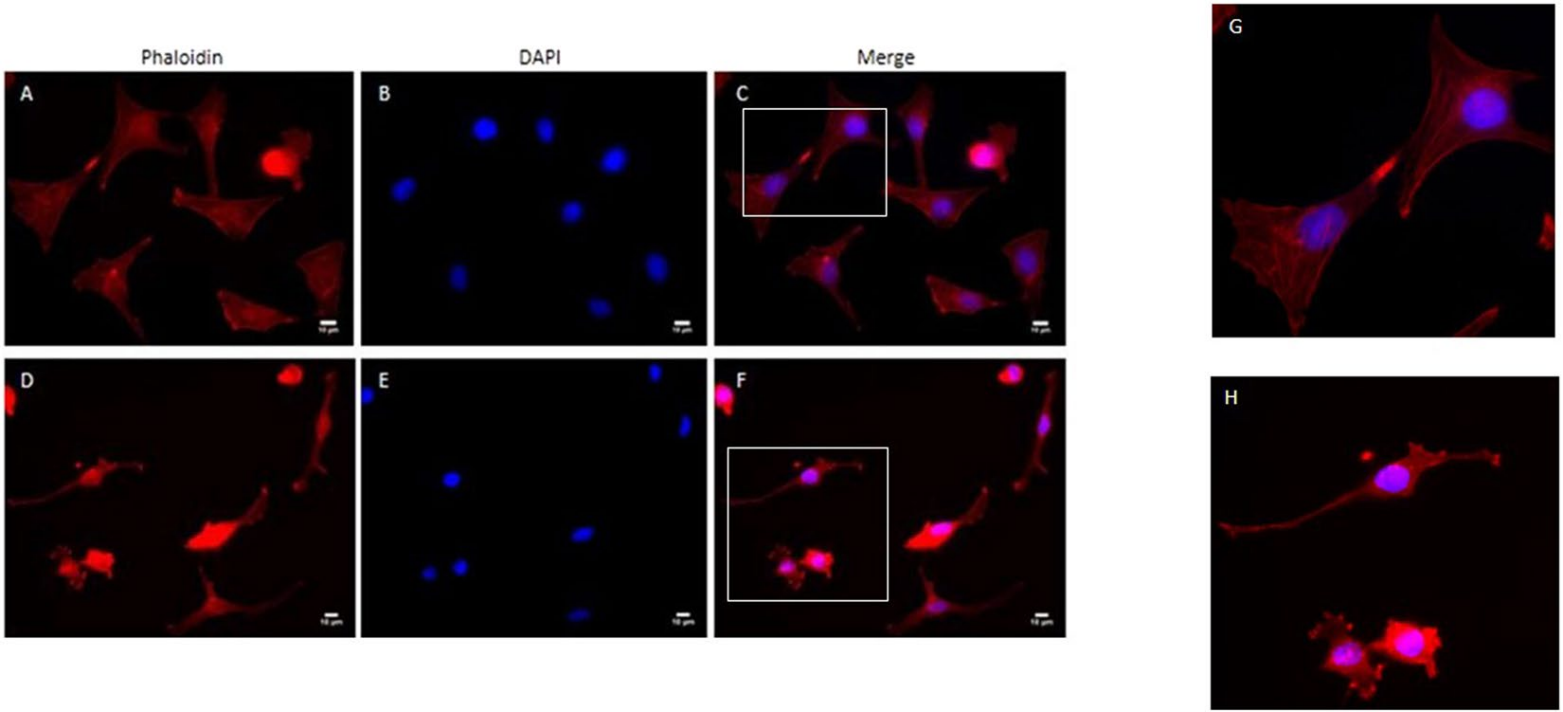
Fasudil treatment disrupts stress fibers in MDA-MB 231 cells. Cells were analysed 24 h after Fasudil treatment by staining with the F-actin probe Texas red-Phalloidin (red, **A**,**D**) and the nuclear stain Dapi (blue, **B**,**E**). Merged images show Phalloidin/Dapi labelling (**C**,**F**). Note that control group (**A**,**B**,**C**) displayed many stress fibers, whereas treatment with 10 μM Fasudil (**D**,**E**,**F**) leads to partial stress fibre disorganization. Panels G and H represents images in higher magnification from areas indicated in panels C and F, respectively. Scale bars represent 50 μm.

Since we found that treatment of MDA-MB 231 cells with 10 µM of Fasudil for 24 h was able to reduce the cell migration without interfering in cell viability (Figs 1 and 2), we decided to use this concentration of Fasudil and duration of treatment for all the subsequent experiments to analyse its effects in cell morphology. Interestingly, we found that Fasudil induces changes in cell morphology. Control cells were well spread over the dishes and displayed many ruffled membranes and lamellipodia, whereas in Fasudil-treated cells membrane protrusions were less prominent and most of the cells displayed spindle shaped morphology with long and fine membrane extensions (Fig. 4A and B). These differences are clearly observed when cells were labelled with phalloidin, which stains filamentous actin, showing stress fiber in control untreated cells. Fasudil treatment induced a partial stress fiber disorganization (Fig. 5A and B).

**Figure 6:**
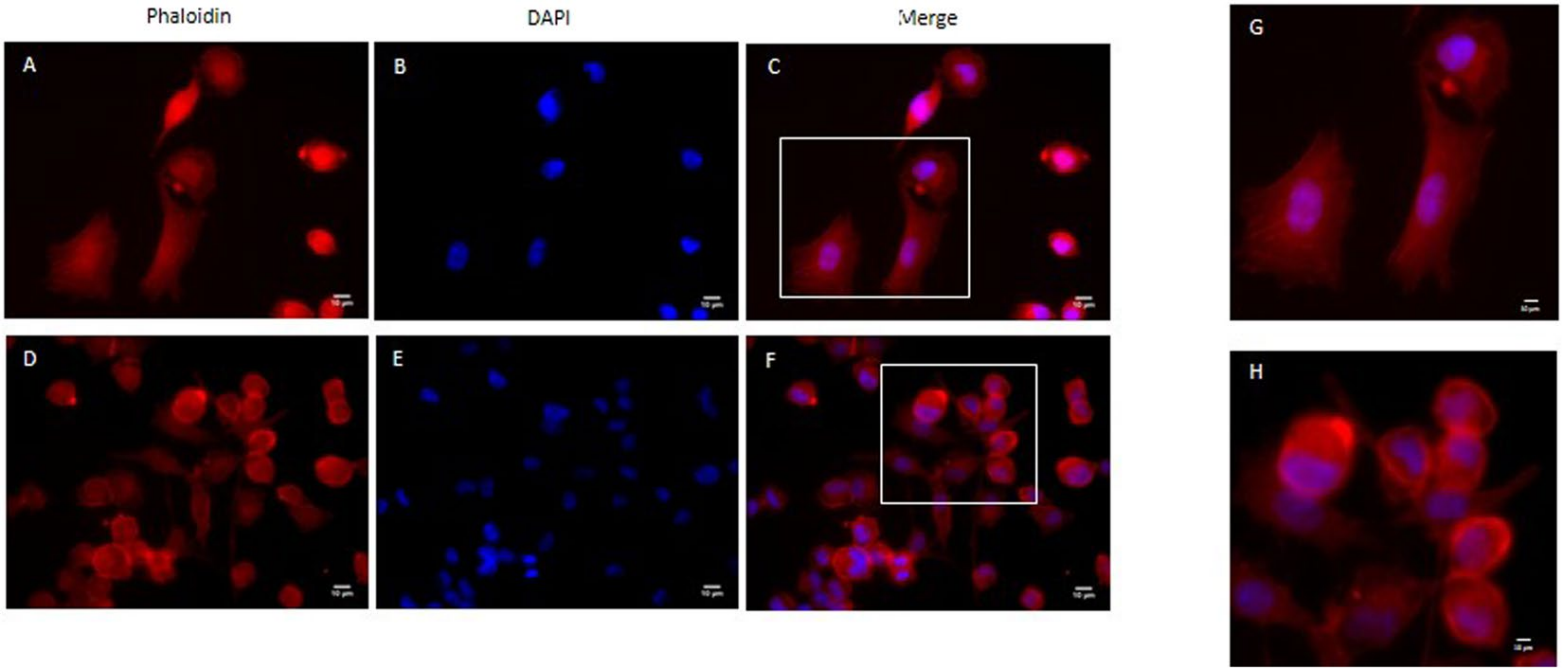
Fasudil treatment disrupts stress fibers in A549 cells. Cells were analysed 24 h after Fasudil treatment (10 μM) by staining with the F-actin probe Texas red-Phalloidin (red, **A**,**C**,**D**, **F**) and the nuclear stain Dapi (**B**,**C**,**E**,**F**). Merged images show Phalloidin/Dapi labelling (**C**,**F**). Note that control group (**A**,**B**,**C**) display many stress fibers, whereas treatment with 10 μM Fasudil (**D**,**E**,**F**) leads to partial stress fibre disorganization. Panels G and H represents images in higher magnification from areas indicated in panels C and F, respectively. Scale bars represent 10 μm.

Same protocol was performed using A549 cell line. Similarly to the results obtained with MDA-MB 231 cells, Fasudil (10 μM) also caused morphological alterations in this tumor cell. Actin disruption with stress fibre disorganization was observed after 24 h incubation (Fig. 6).

### Fasudil-treatment activates the canonical-Wnt pathway in MDA-MB 231 but not in A549 cells

**Figure 7:**
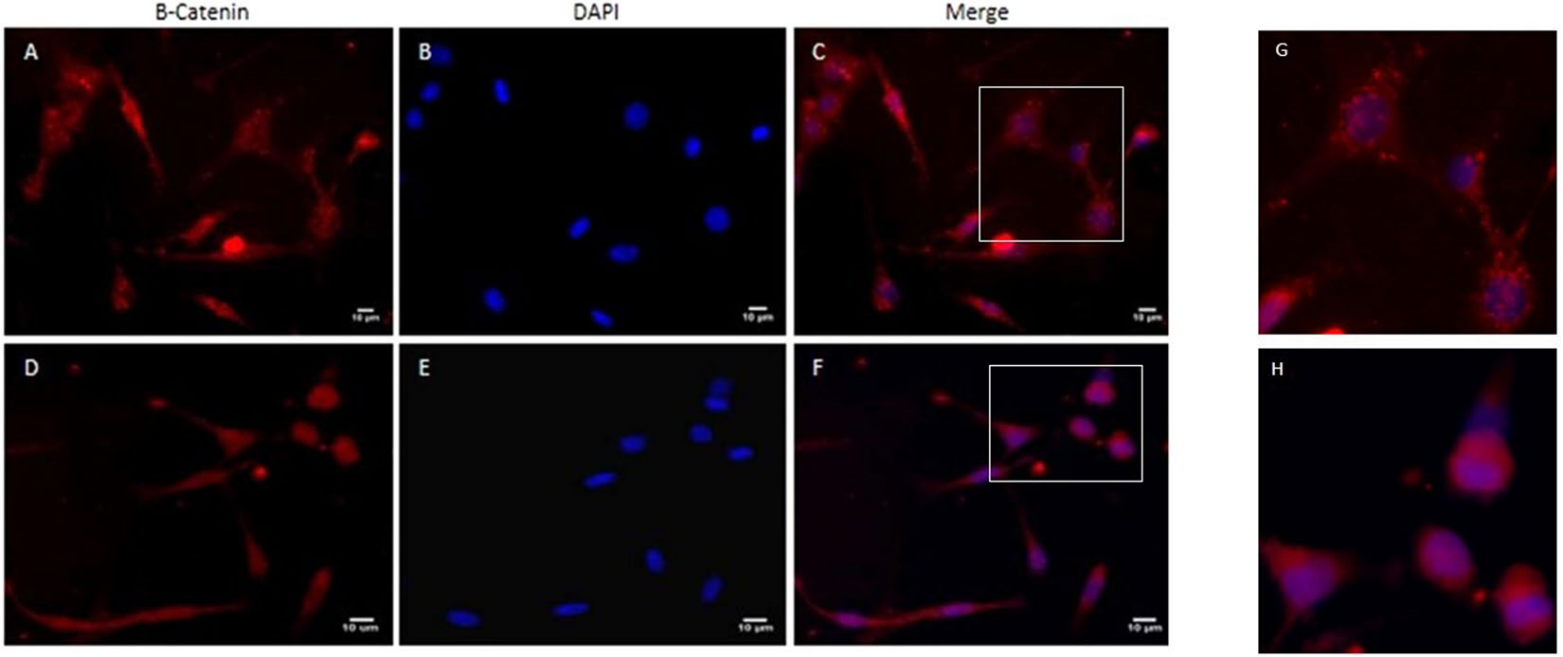
Analysis of the activation of the canonical-Wnt pathway after treatment with Fasudil. MDA-MB 231 cells were grown for 24 h cultures and treated with Fasudil (10 µM) for 24 h. Cells were analysed 24 h after Fasudil treatment by immunofluorescence microscopy for beta-catenin (red, **A**,**D**) and the nuclear stain DAPI (blue, **B**,**E**). Merged images show β-catenin/DAPI (**C**,**F**). Note the distribution of the beta-catenin throughout the cytoplasm of control cells and within the nuclei in cells treated with Fasudil. Panels G and H represents images in higher magnification from areas indicated in panels C and F, respectively. Scale bars represent 10 μm.

To test a possible involvement of the Wnt/beta-catenin pathway in observed effects of Fasudil, we analysed beta-catenin localization using immunofluorescence microscopy. In control cells (Fig. 7A,B,C) beta-catenin was localized in the cytosol (Fig. 7A,C). However, after 24 h of treatment with Fasudil (10 µM) (Fig. 7D,E,F), beta-catenin was found within the nuclei of MDA-MB 231 cells (Fig. 7D,F).

**Figure 8:**
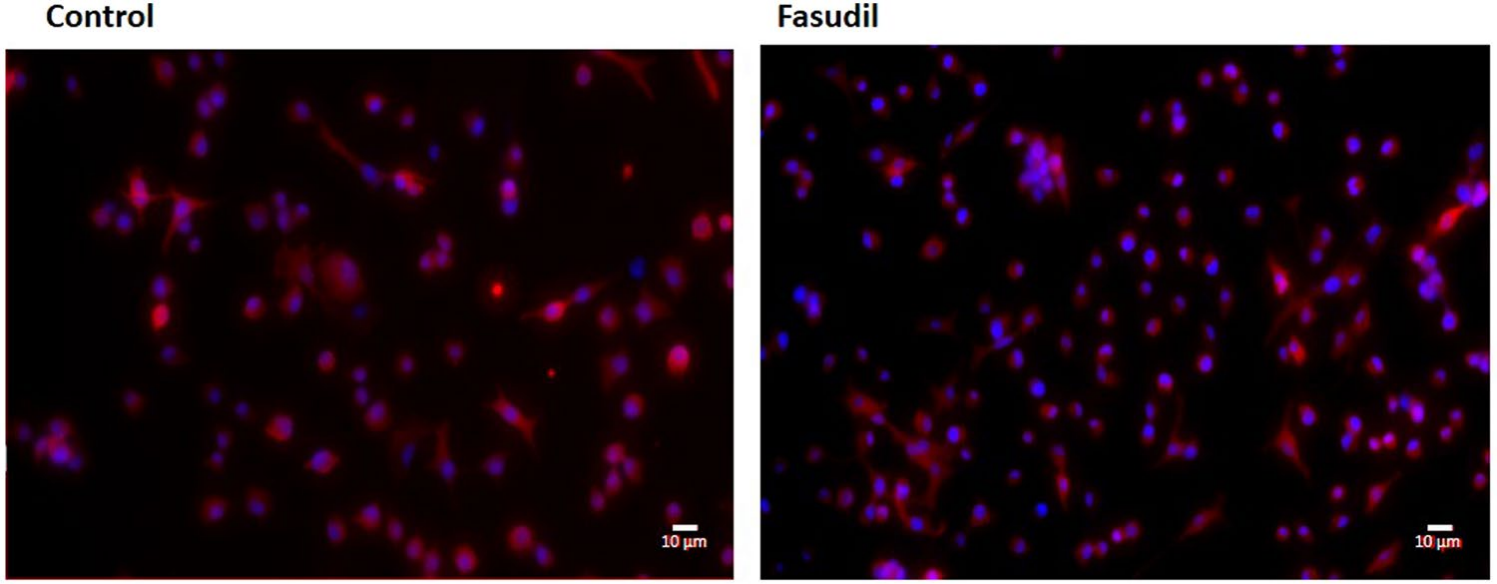
Analysis of the activation of the canonical-Wnt pathway after treatment with Fasudil. A549 cells were grown for 24 h cultures and treated with Fasudil (10 µM) for 24 h. Cells were analysed 24 h after Fasudil treatment by immunofluorescence microscopy for beta-catenin (red) and the nuclear stain DAPI (blue). Merged images show beta-catenin/DAPI. Scale bars represent 10 μm.

When we analysed the activation of the Wnt canonical pathway in the A549 line, we did not observe the translocation of the beta-catenin to the nucleus, suggesting that the reduction of the cell migration in the A549 line is following the non-canonical pathway of Wnt, with alterations in the actin cytoskeleton and consequently in cell morphology (Fig. 8).

## Discussion

It is well known that cancer progression and metastasis require cell motility^11^, and cell migration is a key step in angiogenesis. Fasudil markedly reduced MDA-MB 231 cell migration and lead to partial actin filaments disorganization, resulting in a reduced *in vitro* invasive ability of TNBC MDA-MB 231 cells. The current study showed that Fasudil did not alter human TNBC MDA-MB 231 cell viability at 10 µM. The IC50 concentration for killing MDA-MB 231 cells was higher than 100 μM at 24 and 48 h. Fasudil was found to be effective at reducing migration of the MDA-MB 231 cell line *in vitro*, as evidenced through a cell-based scratch assay. This effect was not cell specific since when we used another tumor cell line originated from lung (A549) similar effects against cell migration was observed. Fasudil also caused the disorganization of stress fibers. In addition, beta-catenin was found within the nuclei which suggests that the Wnt/beta-catenin signalling pathway was activated by Fasudil. These results indicate that Fasudil is an agent in the prevention of metastasis of breast tumor cells of highly aggressive strains such as TNBC MDA-MB 231. Differently, lung tumor cells (A549) did not translocate β-catenin to nucleus suggesting that the reduction in cell migration can occurs due to non-canonical Wnt pathway, resulting alterations in cytoskeleton and consequently in cell morphology.

To the best of our knowledge, this is the first evidence of a correlation between both Wnt pathways (canonical and non-canonical) in MDA-MB 231 cells. This relationship has not yet been well elucidated and our results with both tumor cell lines (from breast and lung) may help to clarify the interaction between these Wnt pathways.

The small GTPases of the Rho family (i.e. RhoA, Rac1 and Cdc42) are known regulators of the actin cytoskeleton^12^. The GTPase RhoA activates ROCK, which is a major regulator of actin cytoskeleton dynamics^13^. Fasudil signalling might change stress fiber organization through the inhibition of ROCK in MDA-MB 231 cells. These results suggest that the ability of Fasudil to affect MDA-MB 231 cells migration, invasion and actin organization are linked to attenuated RhoA/ROCK activation and signalling. These results agree with published studies, showing that the Rho kinase inhibitor Fasudil causes morphologic changes of TNBC MDA-MB 231 cells further supporting the central role of the Rho/Rho kinase pathway in regulating reorganization and assembly of the cytoskeleton^14^.

Previous experiments of our group have shown the involvement of the Wnt/beta-catenin pathway in the modulation of cell migration in MDA-MB 231 cells^9^. Thus, we decided to study the possible involvement of the Wnt/beta-catenin pathway in the reduction in cell migration observed after Fasudil treatment. The canonical Wnt/beta-catenin pathway can regulate transcription factors that control cell movement/invasion. Wnt binds to specific cell-surface receptors Frizzled and this disrupts the destruction complex of beta-catenin which translocate to the nucleus where it activates TCF/Lef1 transcription complex. beta-catenin also promotes cell-to-cell adhesion by accumulating in cell–cell contact sites, namely the adherens junctions. Increased cytoplasmic and nuclear beta-catenin is frequently found in different cancer types, but its impact on the individual tumour pathology can differ strikingly. β-catenin signalling decreases the migration of melanocytes and melanoma cell lines *in vitro* but promotes lung metastases in the NRAS-driven melanoma murine model^15^ and also in a murine model, it has already been observed that ROCK activates beta-catenin and causes epidermal hyperplasia in murine skin by actomyosin contractility and increased epidermal cell proliferation^16^. Also, in liver cancer nuclear beta-catenin is correlated with invasion, enhanced metastasis, poor prognosis and reduced disease-free survival^17,18^. In an earlier study, it was demonstrated that ROCK inhibition induces MCF-7 dormant breast cancer cells to disseminate through the disintegration of cell junctions concomitant with increased cell proliferation, migration and invasion through reduced expression of E-cadherin, beta-catenin, and actin filament bundles at the cell membrane. Interesting, we show here the opposite, ROCK inhibition by Fasudil induces beta-catenin signalling and the inhibition of cell migration of MDA-MB 231 cells. The cell-type specificity or developmental stage of cancer cells might explain the conflicting roles of ROCK in cancer cells. Further studies are needed to clarify the relationship between ROCK and Wnt/beta-catenin during breast tumor cell migration.

## Methods

### Synthesis of Fasudil

**Figure 9:**
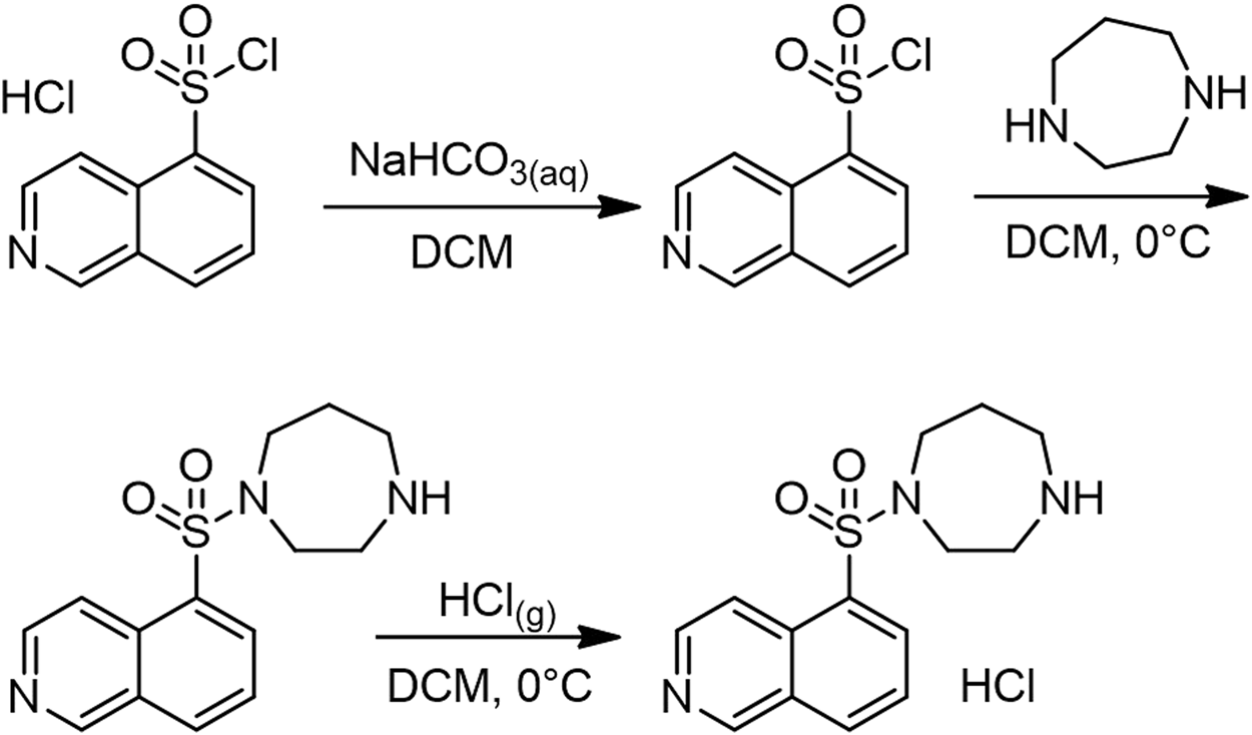
Synthesis of Fasudil hydrochloride.

Fasudil was synthesized as shown in Fig. 9, following the procedure previously described^19^. Isoquinoline sulfonyl chloride hydrochloride was slowly added to a saturated sodium bicarbonate solution. The mixture was kept at constant pH 5-6. The solution was stirred for 30 min and extracted with dichloromethane (DCM). The organic phase was dried with anhydrous sodium sulphate and evaporated under reduced pressure. The residue was dissolved in DCM and dropwise added to a solution of homopiperazine in an ice-cold bath. The resulting mixture was stirred at room temperature for 4 h and solvent was evaporated. The remaining oil was purified by flash chromatography (methanol/ethyl acetate 1/1, V/V). The purified free base was dissolved in DCM and hydrochloric acid (HCl) was bubbled in the ice-cold DCM solution to afford Fasudil hydrochloride. ^1^H NMR (400 MHz, D_2_O) δ: 9.86 (1H, s); 9.04 (1H, d, J = 7 Hz); 8.74–8.79 (3 H, m); 8.17 (1H, t, J = 8Hz); 2.18–2.24 (2H, m); 3.46–3.52 (4H, m); 3.64–3.68 (2H, m); 3.82–3.85 (2H, m); (ESI-MS) [M + H]^+^ m/z: 291.93; HPLC purity (%): 99%. A stock solution of Fasudil was prepared in culture medium (RPMI) and maintained at −20 °C until use. To experiments, aliquots of Fasudil were freshly prepared and used in the same day.

### Cell culture

All cell culture reagents were purchased from Invitrogen (São Paulo, Brazil). The human mammary gland/breast epithelial adenocarcinoma cell line MDA-MB 231 and epithelial cell carcinoma from lung (A549) were obtained from the American Type Culture Collection (ATCC® HTB-26 and CRM-CLL-185, respectively). Cells were routinely grown in RPMI medium containing 10% fetal bovine serum, 1% L-glutamine and 1% penicillin-streptomycin (henceforth called RPMI), in a humidified 5% CO_2_ atmosphere at 37 °C. Cells were cultured up to 70–100% confluences and some cultures were treated with Fasudil, a ROCK-inhibitor, at different concentrations for 24 or 48 h.

### Cell viability assay

Cell viability was determined using 3-(4,5-dimethyl-2-thiazyl)-2,5-diphenyl-2H-tetrazolium bromide (MTT) reagent (Sigma-Aldrich, USA). Briefly, cells were plated at an initial density of 2.5 × 10^4^ cells per well in 96-well plates and incubated for 24 h at 37 °C and 5% CO_2_. After 24 h cultures were treated with Fasudil at a final concentration of 0.1, 1, 10, 50, 100 µM and further incubated for 24 or 48 h. After Fasudil treatment, the supernatant of each well was removed and cells were washed twice with medium. Then, 10 μl of MTT solution (5 mg/ml in RPMI) and 100 μl of medium were added to each well and incubated for 4 h at 37 °C, 5% CO_2_. The resultant formazan crystals were dissolved in dimethyl sulfoxide (100 μl) and absorbance intensities were measured in a microplate reader (FlexStation Reader, Molecular Devices, USA) at 570 nm. All experiments were performed in triplicate, and cell viability was expressed as a percentage relative to the untreated control cells.

### Cell-based scratch assay

Cells were cultured in 24-well culture plates for 24 h up to 90–100% confluence. Scratched wound lines were created with the help of a 200 μl micropipette tip. Wells were washed with RPMI for removal of non-adherent cells. Cells were incubated for 24 h with Fasudil at a final concentration of 0.1, 1, 10, 50, 100 µM. All cell-based scratch assays were performed in the presence of the anti-mitotic reagent cytosine Arabinoside (Arac; Sigma-Aldrich, USA) at a final concentration of 10^−5^ M to inhibit cell proliferation. After Fasudil treatment, the wound areas were observed with an Axiovert 100 microscope (Carl Zeiss, Germany). Images were acquired with an Olympus DP71 digital camera (Olympus, Japan) and the wound area was quantified using Fiji software (based on ImageJ, http://imageJ.nih.gov/ij/) from 3 different experiments.

### Immunofluorescence and digital image acquisition

Cultured cells were fixed with 2% formaldehyde in PBS for 3 min after washing with PBS at 37 °C. Cells were permeabilized with 0.5% Triton X-100 in PBS for 10 min, three times. Cells were incubated with a rabbit polyclonal antibody against beta-catenin (1:50 dilution, Sigma-Aldrich, USA) for 1 hour at 37 °C. After washing with 0.5% Triton X-100 in PBS, cells were incubated with Alexa Fluor 546-conjugated anti-rabbit antibodies (1:200 dilution, Molecular Probes, USA) for 1 hour at 37 °C. To localize F-actin, some specimens were stained with Texas Red-phalloidin (3.3 µM, Molecular Probes, USA) for 20 min at 37 °C. Nuclei were labelled with DAPI (0.1 μg/ml in 0.9% NaCl) and cells were mounted in ProLong Gold antifade reagent (Molecular Probes) and examined with an Axiovert 100 microscope (Carl Zeiss, Germany). Images were acquired with an Olympus DP71 digital camera (Olympus, Japan). Image processing was performed using Fiji software (based on ImageJ, http://imageJ.nih.gov/ij/).

### Statistical analysis

All the values are represented as the means ± standard error. Statistical analysis was performed with one-way ANOVA with Newman-Keuls post-test and statistical significance was defined as *p < 0.05.
